# Supplementary material for: A humanized nanobody phage display library yields potent binders of SARS CoV-2 spike
Source: PLoS One. 2022 Aug 10;17(8):e0272364. doi: 10.1371/journal.pone.0272364 (PMC9365158; doi:10.1371/journal.pone.0272364)
Supplement: S1 Fig — (A) Representative IMAC purification. T—total protein (lysate), L—column load (clarified lysate), FT—column flow through, W—column wash (B) Representative preparative SDS-PAGE after size exclusion column purification (SEC). L—column load. (C) SDS-PAGE of purified nanobodies visualized with Imperial protein stain. (D) Electrospray ionization mass spectrometry (ESI-MS) of a representative purified nanobody. All gels in panels A-C are SDS-PAGE/Coomassie staining with mass of protein standards noted in kDa. Red bars indicate fractions pooled. (DOCX) [file pone.0272364.s001.docx]

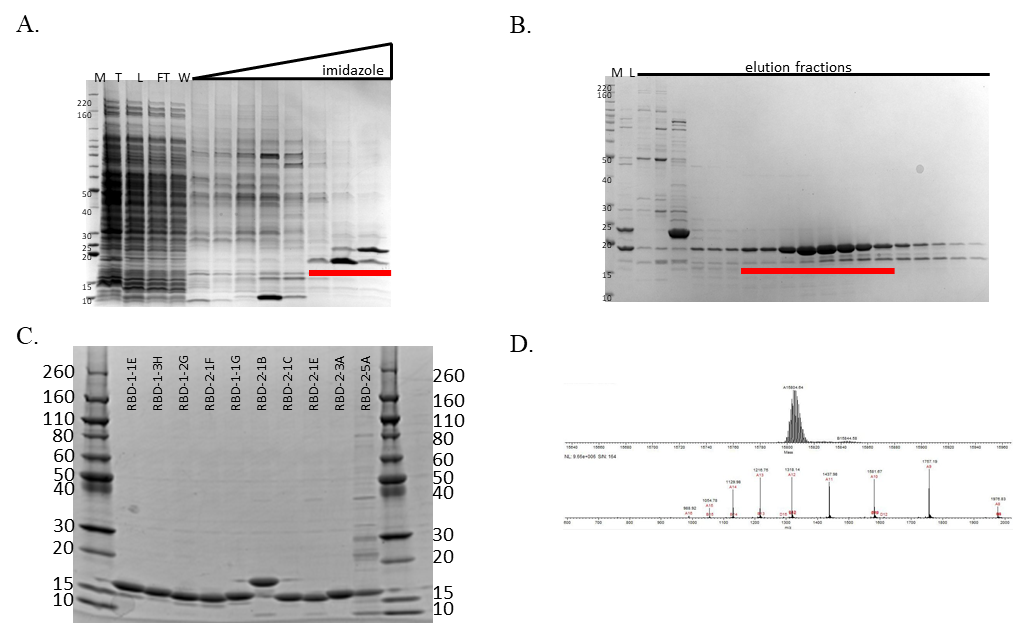


Figure S1: Nanobody purification and quality control. (A) Representative IMAC purification. T – total protein (lysate), L – column load (clarified lysate), FT – column flow through, W – column wash (B) Representative preparative SDS-PAGE after size exclusion column purification (SEC). L – column load. (C) SDS-PAGE of purified nanobodies visualized with Imperial protein stain. (D) Electrospray ionization mass spectrometry (ESI-MS) of a representative purified nanobody. All gels in panels A-C are SDS-PAGE/Coomassie staining with mass of protein standards noted in kDa. Red bars indicate fractions pooled.
