# Supplementary material for: A humanized nanobody phage display library yields potent binders of SARS CoV-2 spike
Source: PLoS One. 2022 Aug 10;17(8):e0272364. doi: 10.1371/journal.pone.0272364 (PMC9365158; doi:10.1371/journal.pone.0272364)
Supplement: S2 Fig — (DOCX) [file pone.0272364.s002.docx]

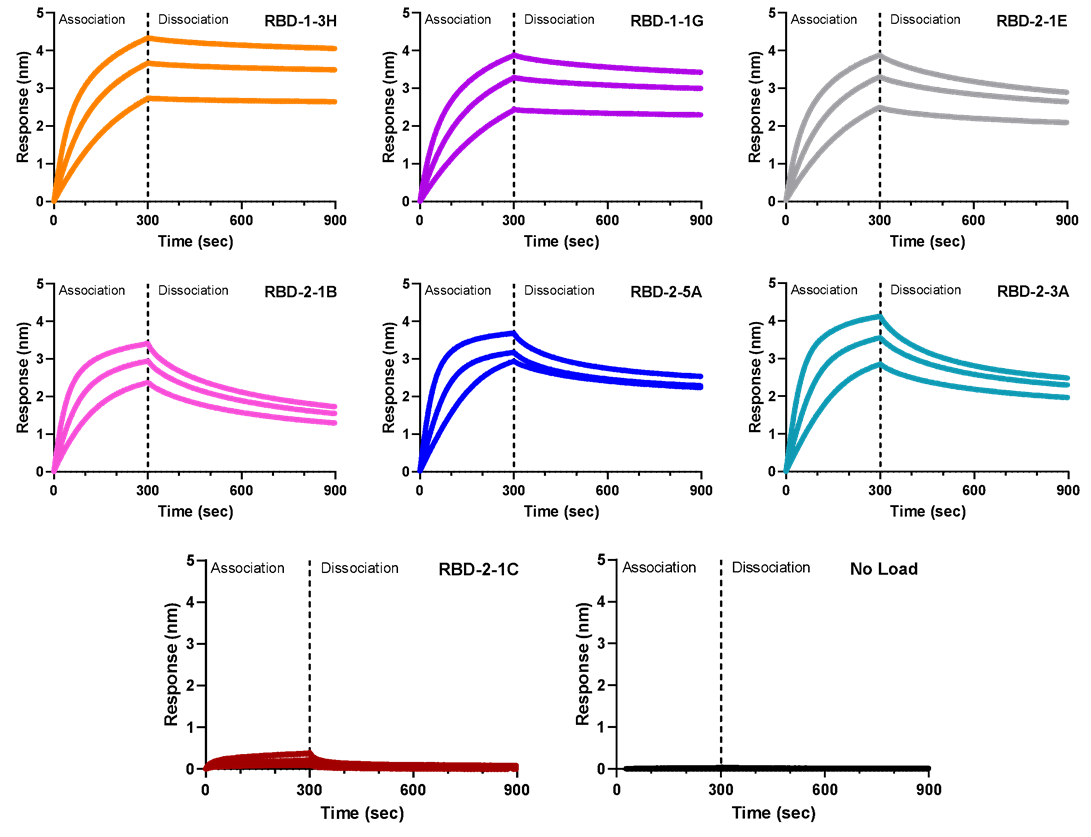


Figure S2: Octet Binding Profiles for immobilized nanobodies binding RBD-mFC for 200 nM, 100 nM and 50 nM concentrations.
