# Supplementary material for: A humanized nanobody phage display library yields potent binders of SARS CoV-2 spike
Source: PLoS One. 2022 Aug 10;17(8):e0272364. doi: 10.1371/journal.pone.0272364 (PMC9365158; doi:10.1371/journal.pone.0272364)
Supplement: S3 Fig — RBD-1-2G, RBD-2-1F and RBD-1-1E also have 25 nM and 12.5 nM conditions. (DOCX) [file pone.0272364.s003.docx]

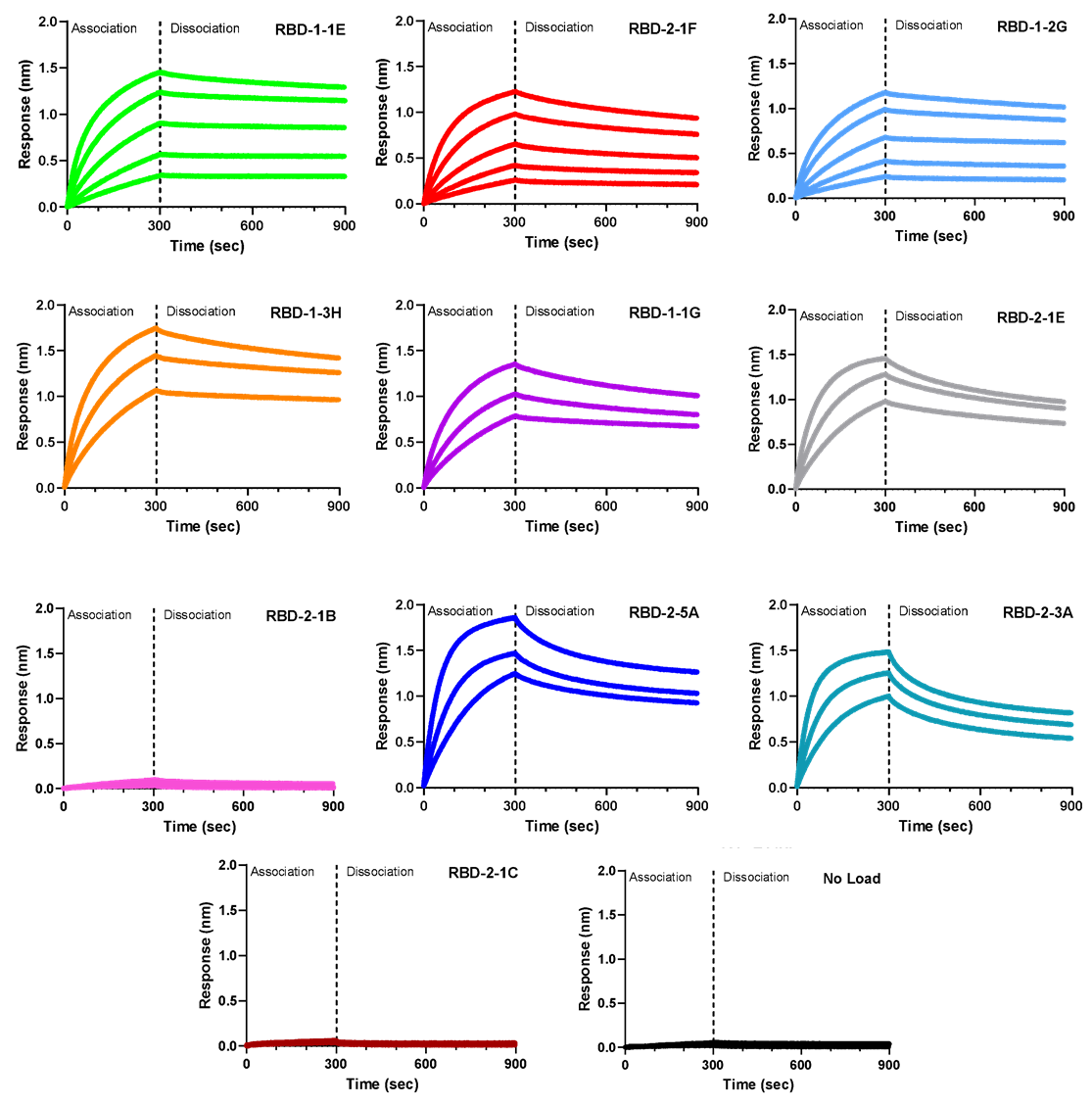


Figure S3: Octet Binding Profiles for immobilized nanobodies binding S1-hFc for 200 nM, 100 nM and 50 nM. RBD-1-2G, RBD-2-1F and RBD-1-1E also have 25 nM and 12.5 nM conditions.
