# Supplementary material for: A humanized nanobody phage display library yields potent binders of SARS CoV-2 spike
Source: PLoS One. 2022 Aug 10;17(8):e0272364. doi: 10.1371/journal.pone.0272364 (PMC9365158; doi:10.1371/journal.pone.0272364)
Supplement: S5 Fig — (A) Representative images for Fc inhibition of QD endoytosis. Representative image montage of ACE2-GFP (yellow) HEK293T cells treated with QD608-RBD (magenta) that was preincubated for 30 minutes with RBD-2-1F-Fc, RBD-1-2G-Fc, RBD-1-1E-Fc, and RBD-2-1E-Fc starting at a concentration of 5 μM or 10 μM. Cells were treated for a total of three hours. Digital Phase Contrast (DPC, cyan) was used to visualize cell bodies. Scale bar, 20 μm. (B-C) Quantification of (B) QD608-RBD and (C) ACE2-GFP using high-content image analysis in each channel. Data was normalized to Optimem I treated cells (100%) and QD608-RBD alone (0%). N = approximately 1600 cells from duplicate wells, representative of three independent experiments. Curves fit using non-linear regression. Error bars indicate S.D. (DOCX) [file pone.0272364.s005.docx]

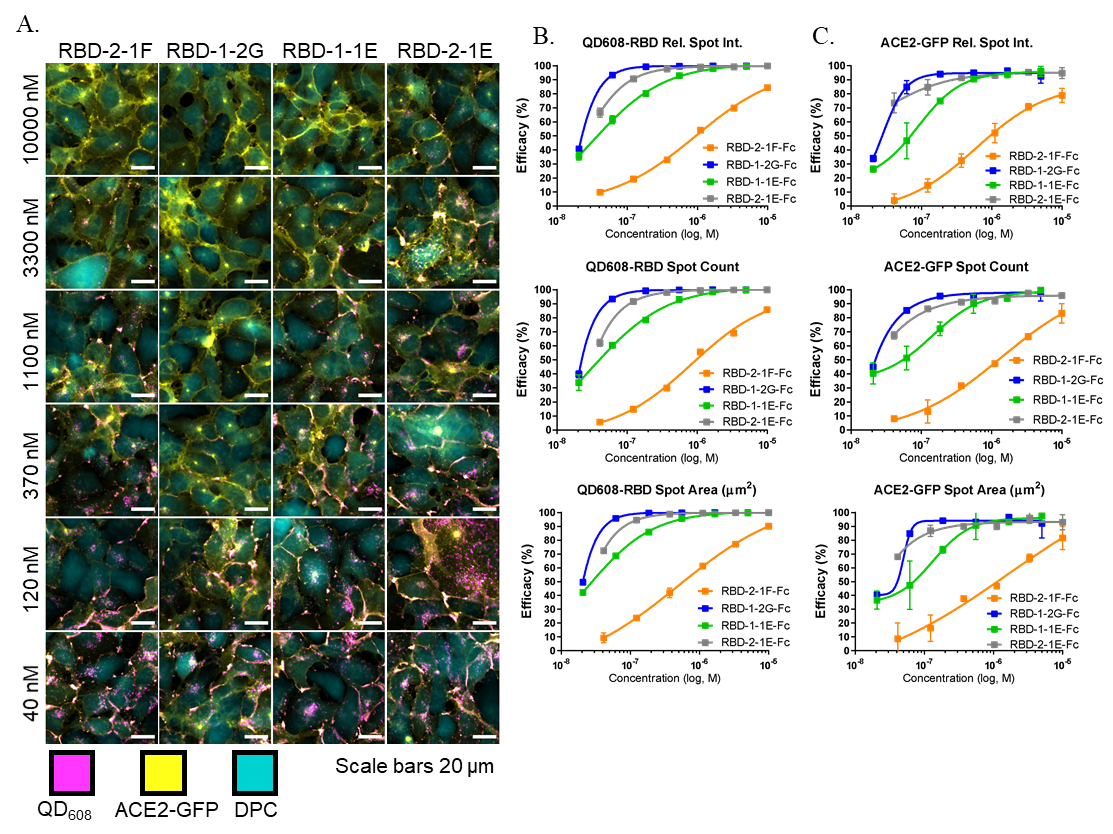


Figure S5: QD ACE2-GFP endocytosis assay with Fc treatment. (A) Representative images for Fc inhibition of QD endoytosis. Representative image montage of ACE2-GFP (yellow) HEK293T cells treated with QD_608_-RBD (magenta) that was preincubated for 30 minutes with RBD-2-1F-Fc, RBD-1-2G-Fc, RBD-1-1E-Fc, and RBD-2-1E-Fc starting at a concentration of 5 µM or 10 µM. Cells were treated for a total of three hours. Digital Phase Contrast (DPC, cyan) was used to visualize cell bodies. Scale bar, 20 µm. (B-C) Quantification of (B) QD_608_-RBD and (C) ACE2-GFP using high-content image analysis in each channel. Data was normalized to Optimem I treated cells (100%) and QD608-RBD alone (0%). N= approximately 1600 cells from duplicate wells, representative of three independent experiments. Curves fit using non-linear regression. Error bars indicate S.D.
