# Supplementary material for: A humanized nanobody phage display library yields potent binders of SARS CoV-2 spike
Source: PLoS One. 2022 Aug 10;17(8):e0272364. doi: 10.1371/journal.pone.0272364 (PMC9365158; doi:10.1371/journal.pone.0272364)
Supplement: S6 Fig — (A-B) Octet binding profiles using either RBD-1-2G-Fc(A) or RBD-2-1F-Fc(B) as load protein. Association of RBD-mFc ranging from 200 nM to 6.25 nM (1:2 serial dilutions) were used for global curve fitting. (C-D) Octet biosensors were loaded with RBD-mFc, then exposed to various concentrations of RBD-1-2G-Fc(C) or RBD-2-1F-Fc(D)(200 nM to 6.25 nM, 1:2 serial dilutions). (E) Calculations from global fit modeling for panels A-D. (DOCX) [file pone.0272364.s006.docx]

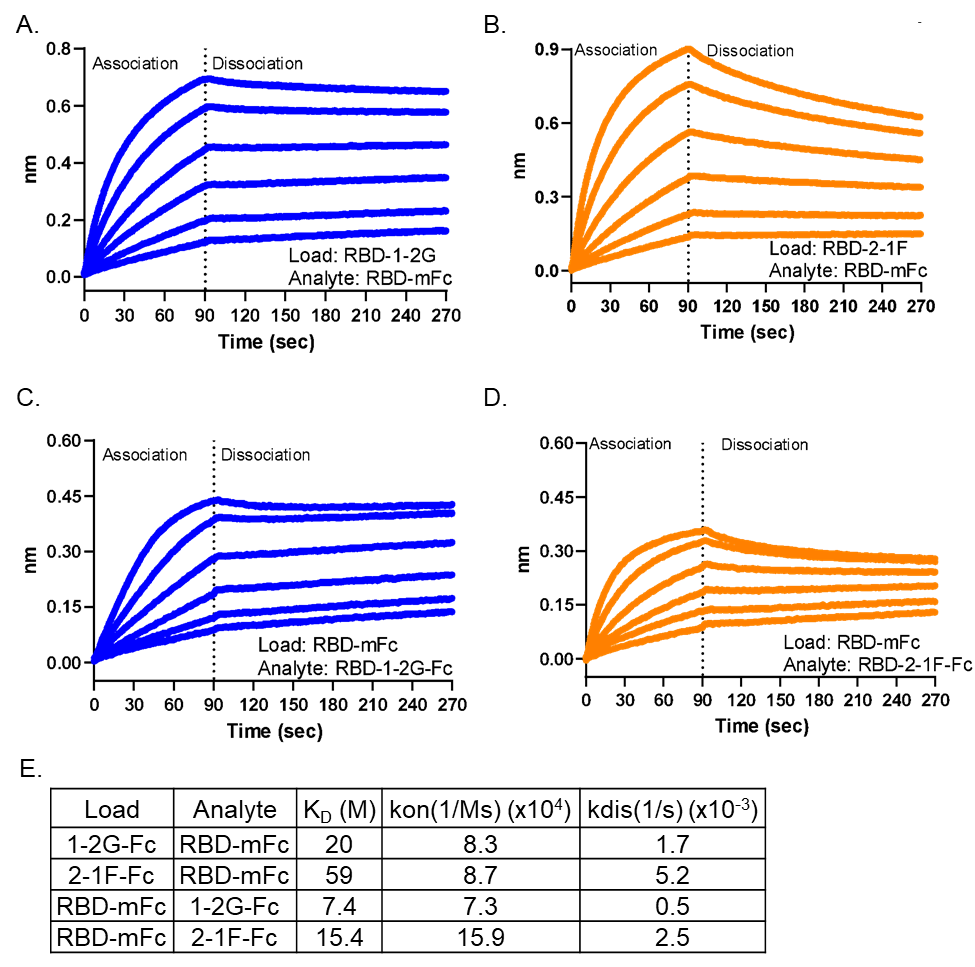


Figure S6: Global Fit curves of 1-2G-Fc and 2-1F-Fc binding to RBD-mFc. (A-B) Octet binding profiles using either RBD-1-2G-Fc(A) or RBD-2-1F-Fc(B) as load protein. Association of RBD-mFc ranging from 200 nM to 6.25 nM (1:2 serial dilutions) were used for global curve fitting. (C-D) Octet biosensors were loaded with RBD-mFc, then exposed to various concentrations of RBD-1-2G-Fc(C) or RBD-2-1F-Fc(D)(200 nM to 6.25 nM, 1:2 serial dilutions). (E) Calculations from global fit modeling for panels A-D.
