# Supplementary material for: A humanized nanobody phage display library yields potent binders of SARS CoV-2 spike
Source: PLoS One. 2022 Aug 10;17(8):e0272364. doi: 10.1371/journal.pone.0272364 (PMC9365158; doi:10.1371/journal.pone.0272364)
Supplement: S7 Fig — SARS-CoV-2 pseudotyped particle entry assay for Nanobody(A), Fc(B) and Trimer(C) formats. (DOCX) [file pone.0272364.s007.docx]

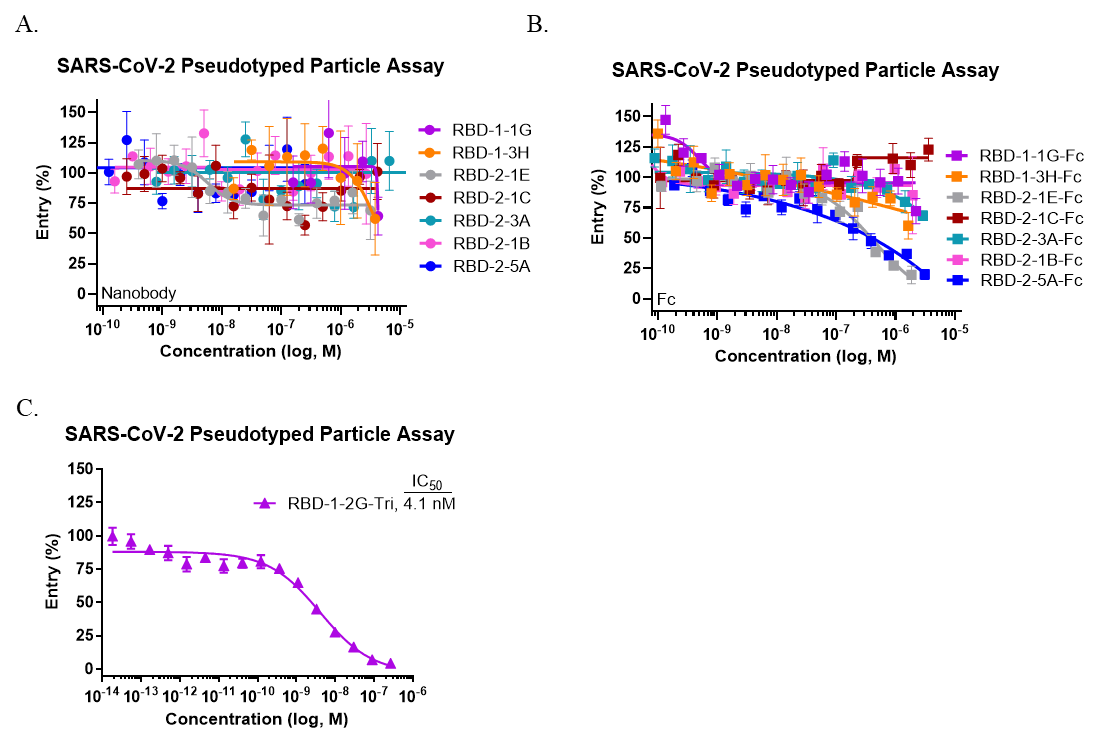
Figure S7: SARS-CoV-2 Pseudotyped particles assay for non-blockers and the trimer. SARS-CoV-2 pseudotyped particle entry assay for Nanobody(A), Fc(B) and Trimer(C) formats.
