# Supplementary material for: A humanized nanobody phage display library yields potent binders of SARS CoV-2 spike
Source: PLoS One. 2022 Aug 10;17(8):e0272364. doi: 10.1371/journal.pone.0272364 (PMC9365158; doi:10.1371/journal.pone.0272364)
Supplement: S8 Fig — (A) Human membrane proteome array (MPA) results identified during a cross-reactivity screen. (B) Follow-up validation screen to determine the reactivity of MIEF1 protein to RBD-1-2G. (DOCX) [file pone.0272364.s008.docx]

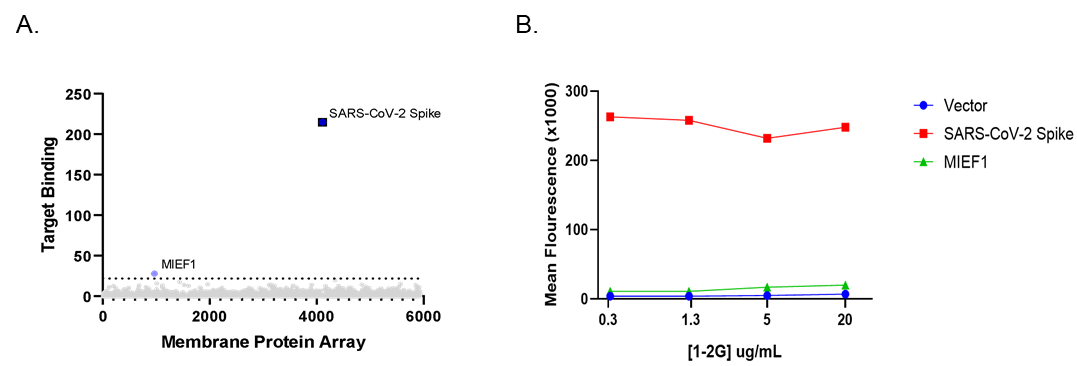


Figure S8: (A-B) Cross-reactivity screen of RBD-1-2G. (A) Human membrane proteome array (MPA) results identified during a cross-reactivity screen. (B) Follow-up validation screen to determine the reactivity of MIEF1 protein to RBD-1-2G.
