# Supplementary material for: A humanized nanobody phage display library yields potent binders of SARS CoV-2 spike
Source: PLoS One. 2022 Aug 10;17(8):e0272364. doi: 10.1371/journal.pone.0272364 (PMC9365158; doi:10.1371/journal.pone.0272364)
Supplement: S9 Fig — RBD-1-2G vs a lyophilized reconstituted sample were tested for their ability to inhibit live virus infection. Titration curves with a top concentration of 15.2 μM for the untreated RBD-1-2G and 15.8 μM for the reconstituted lyophilized RBD-1-2G sample. Samples were diluted 1:2 in dPBS before being used for the viral assay. Technical replicates are n = 3 per concentration, all error bars represent S.D. (DOCX) [file pone.0272364.s009.docx]

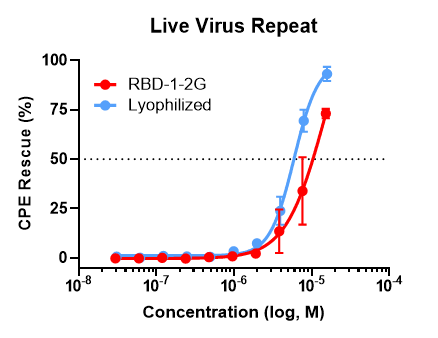


Figure S9: Effect of lyophilization on RBD-1-2G. RBD-1-2G vs a lyophilized reconstituted sample were tested for their ability to inhibit live virus infection. Titration curves with a top concentration of 15.2 µM for the untreated RBD-1-2G and 15.8 µM for the reconstituted lyophilized RBD-1-2G sample. Samples were diluted 1:2 in dPBS before being used for the viral assay. Technical replicates are n = 3 per concentration, all error bars represent S.D.
