# Supplementary material for: A humanized nanobody phage display library yields potent binders of SARS CoV-2 spike
Source: PLoS One. 2022 Aug 10;17(8):e0272364. doi: 10.1371/journal.pone.0272364 (PMC9365158; doi:10.1371/journal.pone.0272364)
Supplement: S10 Fig — (A) Cryo-EM analysis of the 1-1E nanobody complexed with the RBD (up state). (B) Model of RBD-1-2G binding overlaps with the ACE2 binding site, while RBD-1-1G fails to inhibit binding. (C) Overlap of RBD-2-1F and RBD-1-2G suggesting similar epitopes being targeted by ‘Group 1’ binders. (DOCX) [file pone.0272364.s010.docx]

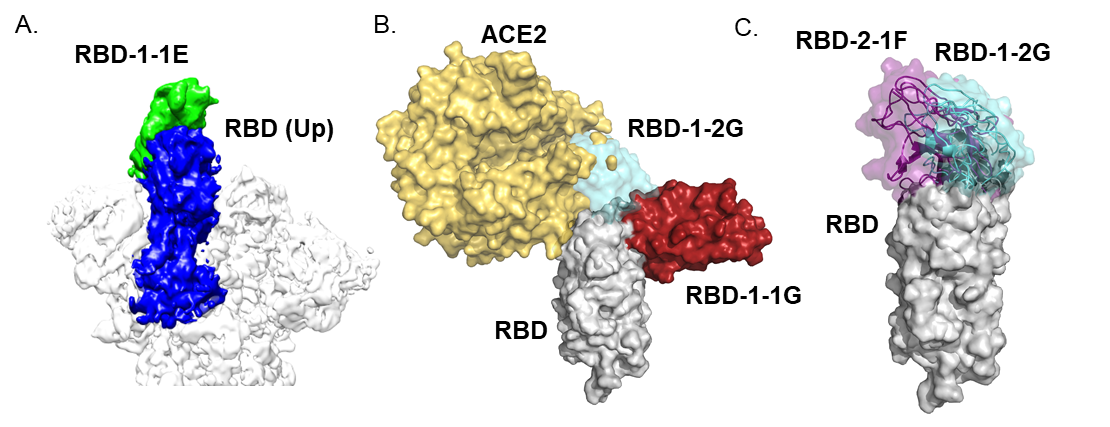


Figure S10: Atomic Fit Models of the RBD/ACE2/Nanobody interactions. (A) Cryo-EM analysis of the 1-1E nanobody complexed with the RBD (up state). (B) Model of RBD-1-2G binding overlaps with the ACE2 binding site, while RBD-1-1G fails to inhibit binding. (C) Overlap of RBD-2-1F and RBD-1-2G suggesting similar epitopes being targeted by ‘Group 1’ binders.
