# Supplementary material for: A humanized nanobody phage display library yields potent binders of SARS CoV-2 spike
Source: PLoS One. 2022 Aug 10;17(8):e0272364. doi: 10.1371/journal.pone.0272364 (PMC9365158; doi:10.1371/journal.pone.0272364)
Supplement: S11 Fig — (DOCX) [file pone.0272364.s011.docx]

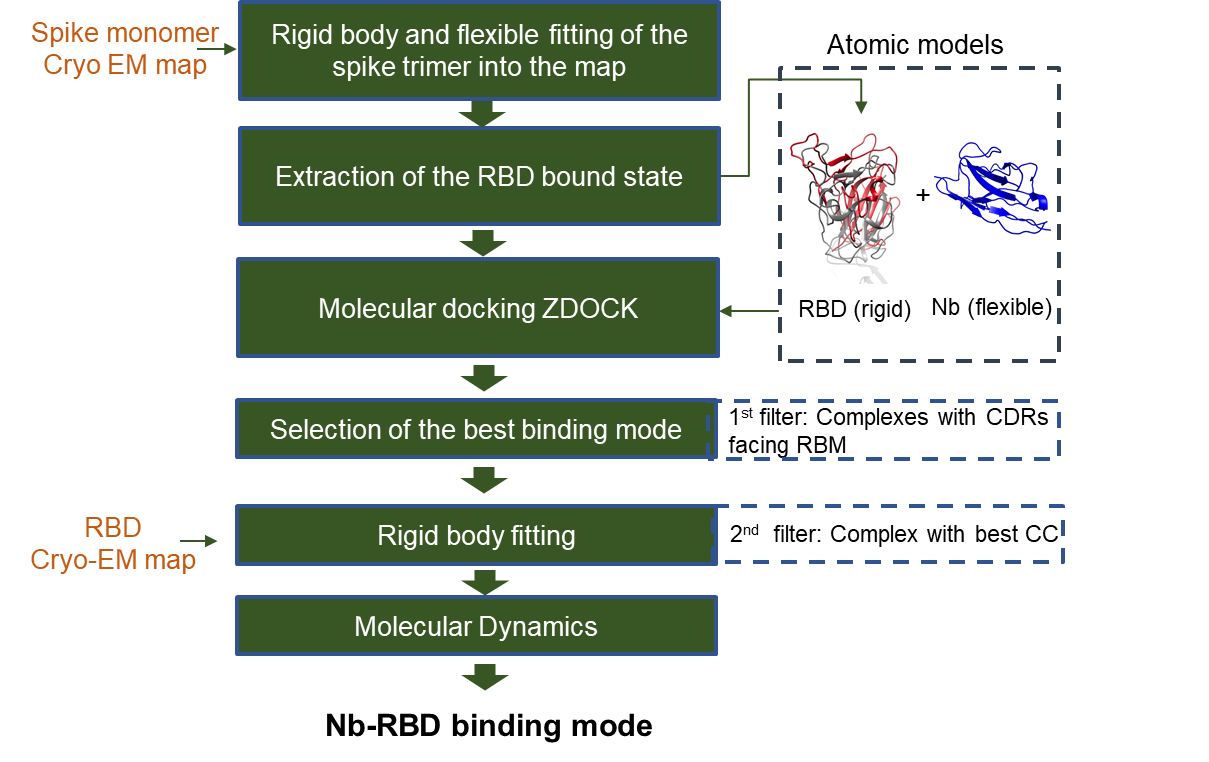


Figure S11: Workflow describing the combination of computational techniques and Cryo-EM applied to identify Nb-RBD binding mode details.
