# Supplementary material for: A humanized nanobody phage display library yields potent binders of SARS CoV-2 spike
Source: PLoS One. 2022 Aug 10;17(8):e0272364. doi: 10.1371/journal.pone.0272364 (PMC9365158; doi:10.1371/journal.pone.0272364)
Supplement: S12 Fig — (DOCX) [file pone.0272364.s012.docx]

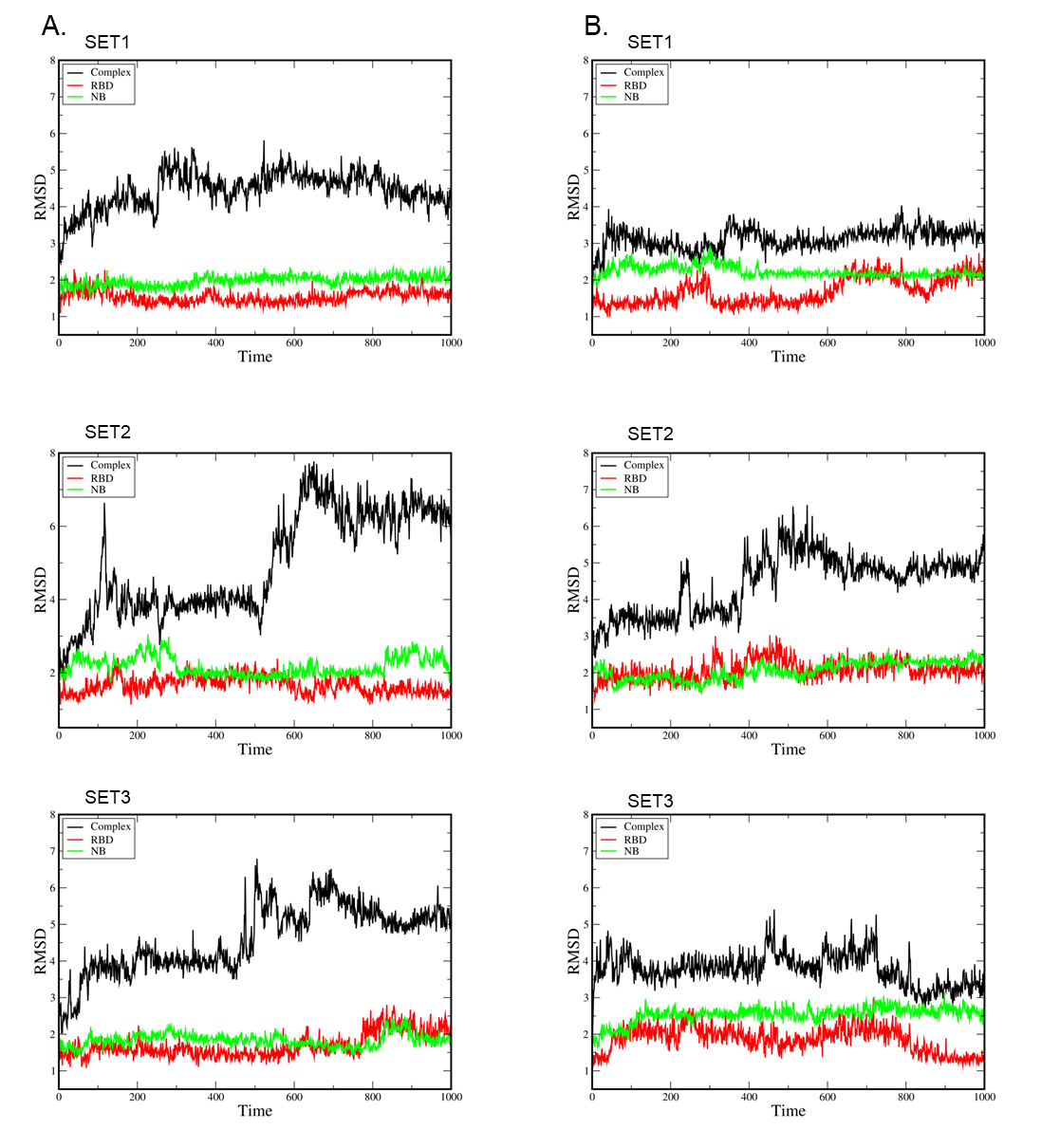


Figure S12: Root mean square deviation (RMSD) of the MD triplicates per column for RBD-1-2G in complex (A) with the WT RBD and (B) with B.1.1.7 RBD showed that the systems were stable.
