# Supplementary material for: A humanized nanobody phage display library yields potent binders of SARS CoV-2 spike
Source: PLoS One. 2022 Aug 10;17(8):e0272364. doi: 10.1371/journal.pone.0272364 (PMC9365158; doi:10.1371/journal.pone.0272364)
Supplement: S13 Fig — Root mean square fluctuation (RMSF) for the (A) WT RBD residues and (B) B.1.1.7 RBD residues, showing the same pattern of variations. Both graphs show two main peaks correspondent to the residues 355–375 and 470–490. (C) The fluctuation of the residues of the RBD-1-2G in complex with the WT RBD and (D) with the B.1.1.7 RBD. (DOCX) [file pone.0272364.s013.docx]

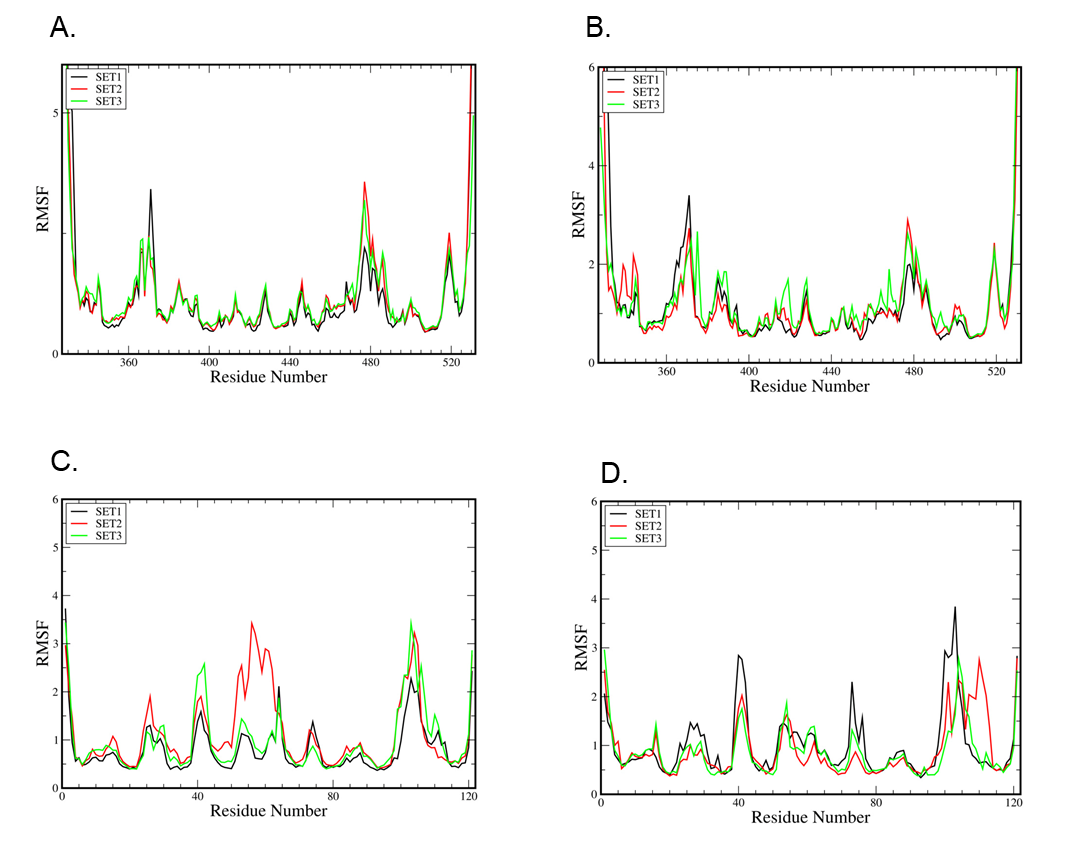


Figure S13: Root mean square fluctuation (RMSF) for the (A) WT RBD residues and (B) B.1.1.7 RBD residues, showing the same pattern of variations. Both graphs show two main peaks correspondent to the residues 355-375 and 470-490. (C) The fluctuation of the residues of the RBD-1-2G in complex with the WT RBD and (D) with the B.1.1.7 RBD.
