# Supplementary material for: A humanized nanobody phage display library yields potent binders of SARS CoV-2 spike
Source: PLoS One. 2022 Aug 10;17(8):e0272364. doi: 10.1371/journal.pone.0272364 (PMC9365158; doi:10.1371/journal.pone.0272364)
Supplement: S14 Fig — (DOCX) [file pone.0272364.s014.docx]

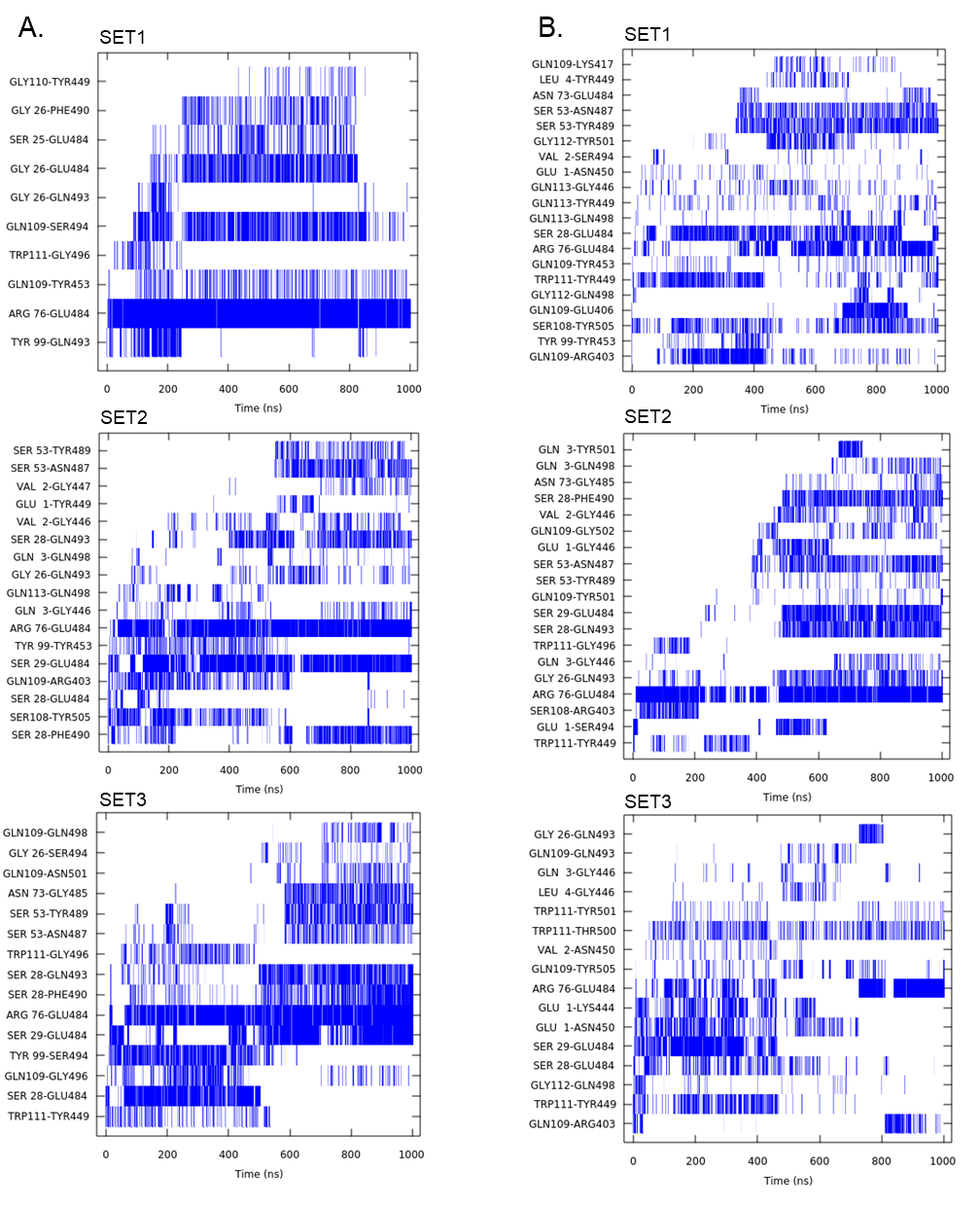


Figure S14: Hydrogen bonds and salt bridge interactions per time of the MD simulations in triplicate for the systems (per column) containing RBD-1-2G with (A) WT RBD and (B) with B.1.1.7 RBD.
