# Supplementary material for: A humanized nanobody phage display library yields potent binders of SARS CoV-2 spike
Source: PLoS One. 2022 Aug 10;17(8):e0272364. doi: 10.1371/journal.pone.0272364 (PMC9365158; doi:10.1371/journal.pone.0272364)
Supplement: S15 Fig — Free energy of binding for RBD-1-2G (A) Total free energy of binding each residue in the WT and B.1.17 variant RBD when bound by RBD-1-2G. (B) Heatmap showing the free binding energy for the WT and B.1.1.7 RBD in complex with RBD-1-2G. (DOCX) [file pone.0272364.s015.docx]

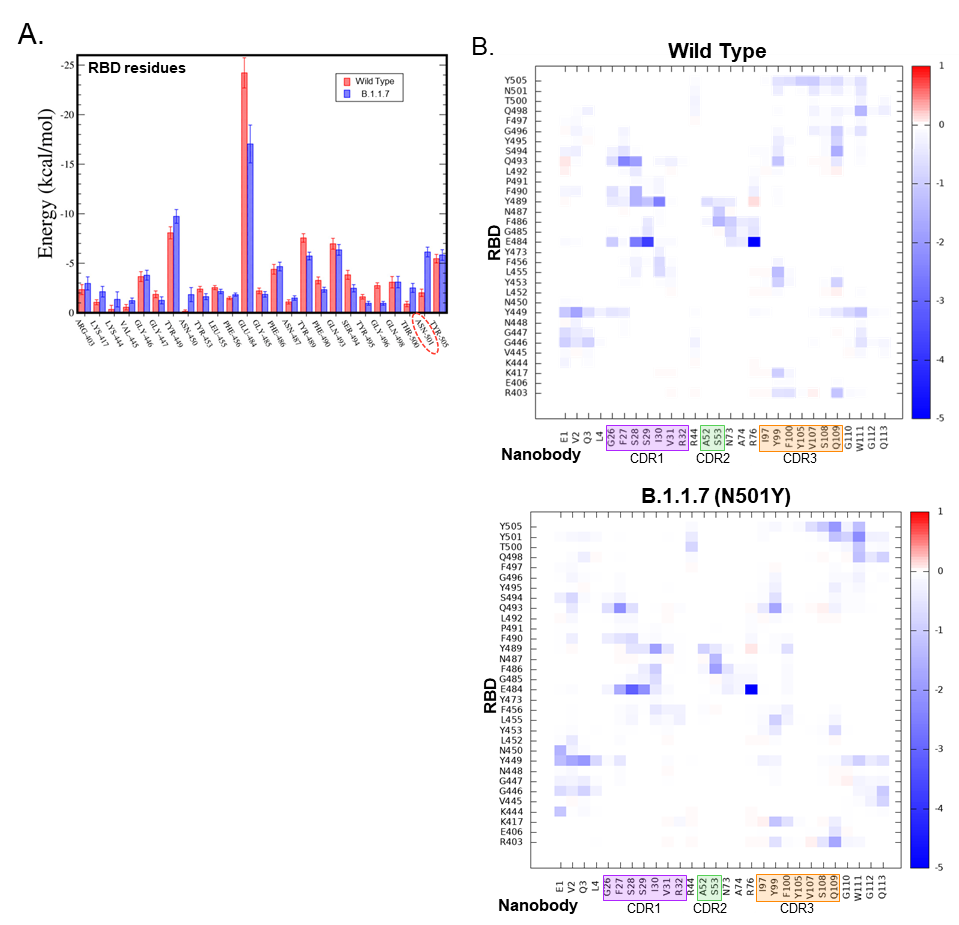


Figure S15: Free energy of binding for RBD-1-2G (A) Total free energy of binding each residue in the WT and B.1.17 variant RBD when bound by RBD-1-2G. (B) Heatmap showing the free binding energy for the WT and B.1.1.7 RBD in complex with RBD-1-2G.
