# Supplementary material for: A humanized nanobody phage display library yields potent binders of SARS CoV-2 spike
Source: PLoS One. 2022 Aug 10;17(8):e0272364. doi: 10.1371/journal.pone.0272364 (PMC9365158; doi:10.1371/journal.pone.0272364)
Supplement: S16 Fig — (A) Response value at the end of the association phase by RBD-1-2G-Fc to various SAR-CoV-2 mutant S1 proteins. (B) Response value at the end of the association phase by RBD-1-2G-Fc to various SAR-CoV-2 mutant extracellular domains. (DOCX) [file pone.0272364.s016.docx]

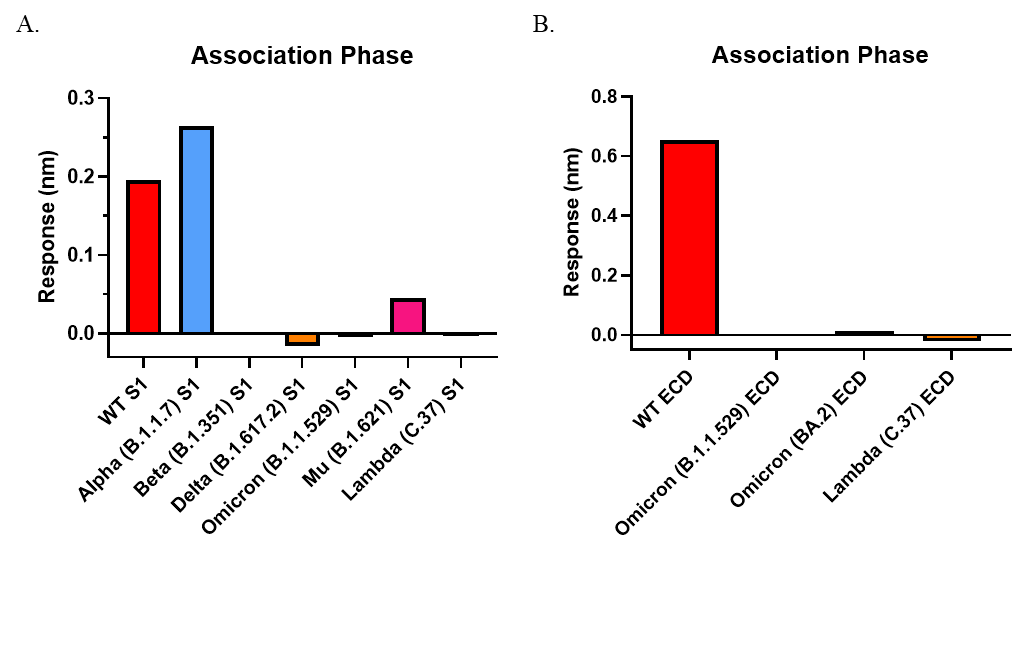


Figure S16: Bio-layer interferometry of immobilized RBD-1-2G-Fc to SARS-CoV-2 spike variants. (A) Response value at the end of the association phase by RBD-1-2G-Fc to various SAR-CoV-2 mutant S1 proteins. (B) Response value at the end of the association phase by RBD-1-2G-Fc to various SAR-CoV-2 mutant extracellular domains.
