# Supplementary material for: A humanized nanobody phage display library yields potent binders of SARS CoV-2 spike
Source: PLoS One. 2022 Aug 10;17(8):e0272364. doi: 10.1371/journal.pone.0272364 (PMC9365158; doi:10.1371/journal.pone.0272364)
Supplement: S17 Fig — Cryo-EM structure of the (A) APO state WT SARS-CoV-2 S-protein in side and top views showing the RBD in the 1 Up, 2 down state. (B) B.1.1.7 SARS-CoV-2 S-protein bound to RBD-1-2G in side and top views showing the RBD in the partially down state. (DOCX) [file pone.0272364.s017.docx]

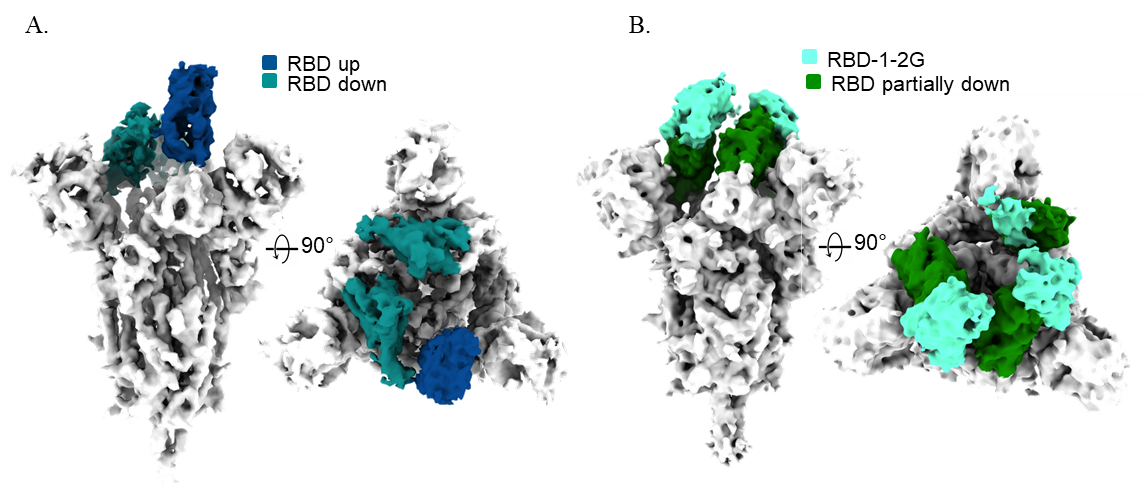


Figure S17: Cryo-EM structure of the (A) APO state WT SARS-CoV-2 S-protein in side and top views showing the RBD in the 1 Up, 2 down state. (B) B.1.1.7 SARS-CoV-2 S-protein bound to RBD-1-2G in side and top views showing the RBD in the partially down state.
