# Supplementary material for: A humanized nanobody phage display library yields potent binders of SARS CoV-2 spike
Source: PLoS One. 2022 Aug 10;17(8):e0272364. doi: 10.1371/journal.pone.0272364 (PMC9365158; doi:10.1371/journal.pone.0272364)
Supplement: S1 Table — (DOCX) [file pone.0272364.s018.docx]

| **Nanobody** | **CDR1 Region** | | **CDR2 Region** | | | **CDR3 Region** | **Sequence Enrichment** |
| --- | --- | --- | --- | --- | --- | --- | --- |
|  | Lead | Variable | Lead | Variable | Tail | Variable |  |
| RBD-1-3H | GNIS | ADAH | IT | NP | GGNTN | SLLFKRNELSNRWT | 8 / 48 (16.7%) |
| RBD-1-1E | GYIS | RSVF | IS | PD | GANTN | IIRYVASNNTEDV | 6 / 48 (12.5%) |
| RBD-1-2G | GFSS | IVY | ID | AS | GSTTN | IAYFTSPEYVVSQG | 6 / 48 (12.5%) |
| RBD-1-1G | GSIS | EYKH | IT | NP | GSNTN | VLFPKTATKNVGWE | 6 / 48 (12.5%) |
| RBD-1-2E | GNIS | AHGV | ID | RP | GANTN | GYTWVAQTQHYSWE | 1 / 48 (2.1%) |
| RBD-2-1B | GYIS | VTGI | IT | VY | GTNTY | KLKSDPVNEPSPEE | 26 / 95 (27.4%) |
| RBD-2-1F | GYIS | Q | ID | QD | GTSTN | ANFLVPGQLRKI | 25 / 95 (26.3%) |
| RBD-2-5A | GNIS | RIVY | IA | AQ | GTSTN | LHTYVAAEKIVEQH | 8 / 95 (8.4%) |
| RBD-2-1C | GYIS | SVSS | IT | AL | GTSTY | GWDFTNAKGVIHET | 7 / 95 (7.4%) |
| RBD-2-1E | GTIS | HETF | ID | AS | GTNTN | VAYFPVLTTQVISL | 5 / 95 (5.3%) |
| RBD-2-3A | GSIS | QAFI | IS | TE | GSSTN | LRKFQPDIQLTQWV | 4 / 95 (4.2%) |
| RBD-2-3H | GSIS | SHEQ | IT | FI | GGNTY | ALIREDSLSKTHWE | 2 / 95 (2.1%) |
| RBD-2-2C | GTIF | HEVW | ID | VI | GTSTY | SGVHQGRRHVKFLV | 1 / 95 (1.1%) |

Supplemental Table 1: Nanobody complementarity-determining regions (CDRs) sequences and phage enrichment stats
