# Supplementary material for: A humanized nanobody phage display library yields potent binders of SARS CoV-2 spike
Source: PLoS One. 2022 Aug 10;17(8):e0272364. doi: 10.1371/journal.pone.0272364 (PMC9365158; doi:10.1371/journal.pone.0272364)
Supplement: S2 Table — (DOCX) [file pone.0272364.s019.docx]

| **Data set** | **Voltage (KeV)** | **Mag (kX)** | **Pixel Size (Å)** | **Underfocus Range (Å)** | **Exposure Rate (e/px/s)** | **Total Dose** | **Movies Collected** |
| --- | --- | --- | --- | --- | --- | --- | --- |
| **RBD-1-2G** | 300 | 130 | 0.532 (SR) | 1.2 – 2.4 | 8 | 60 | 4230 |
| **RBD-1-1G** | 200 | 36 | 1.187 (CM) | 1.2 – 2.2 | 8 | 60 | 2743 |
| **RBD-1-3H** | 200 | 36 | 1.187 (CM) | 1.5 – 2.8 | 8 | 60 | 4977 |
| **RBD-2-1F** | 200 | 36 | 1.187 (CM) | 1.2 – 2.8 | 8 | 60 | 3347 |
| **RBD-2-1B** | 200 | 36 | 1.187 (CM) | 1.4 – 2.4 | 8 | 60 | 1693 |
| **RBD-2-3A** | 200 | 36 | 1.187 (CM) | 1.5 – 2.2 | 8 | 60 | 1755 |

Supplemental Table 2: Microscope parameters used to collect Cryo-EM data of the nanobody complexes.
