# Supplementary material for: A humanized nanobody phage display library yields potent binders of SARS CoV-2 spike
Source: PLoS One. 2022 Aug 10;17(8):e0272364. doi: 10.1371/journal.pone.0272364 (PMC9365158; doi:10.1371/journal.pone.0272364)
Supplement: S3 Table — (DOCX) [file pone.0272364.s020.docx]

| **Complex** | **Interactions** | **Distance (Å)** |
| --- | --- | --- |
| RBD (WT)  + RBD-1-2G | E484-R76 (1) | 2.8 |
|  | E484-R76 (2) | 2.6 |
|  | E484-S25 | 2.7 |
|  | Y489-S29 | 3.3 |
|  | N501-Y99 | 3.1 |
|  | N501-Q498 | 3.2 |
|  | N501-G496 | 3.0 |
| RBD (B.1.1.7)  + RBD-1-2G | E484-R76 (1) | 3.0 |
|  | E484-R76 (2) | 2.7 |
|  | E484-S28 | 2.8 |
|  | W111-T500 | 3.3 |
|  | Y501-G112 | 2.7 |
|  | Y501-Q498 | 3.2 |

Supplemental Table 3: Distance of most relevant intra- and inter-molecular hydrogen bonds in complexes containing RBD-1-2-G with RBD (WT) or RBD (B.1.1.7).
