# Supplementary material for: A humanized nanobody phage display library yields potent binders of SARS CoV-2 spike
Source: PLoS One. 2022 Aug 10;17(8):e0272364. doi: 10.1371/journal.pone.0272364 (PMC9365158; doi:10.1371/journal.pone.0272364)
Supplement: S1 Methods — (DOCX) [file pone.0272364.s021.docx]

A humanized nanobody phage display library yields

potent binders of SARS CoV-2 spike

Ying Fu^1¶^, Juliana da Fonseca Rezende e Mello^2¶^, Bryan D. Fleming^1¶^, Alex Renn^1¶^, Catherine Z. Chen^1^, Xin Hu^1^, Miao Xu^1^, Kirill Gorshkov^1^, Quinlin Hanson^1^, Wei Zheng^1^, Emily M. Lee^1^, Lalith Perera^2^, Robert Petrovich^2^, Manisha Pradhan^1^, Richard T. Eastman^1^, Zina Itkin^1^, Thomas B. Stanley^2^, Allen Hsu^2^, Venkata Dandey^2^, Kedar Sharma^2^, William Gillette^3^, Troy Taylor^3^, Nitya Ramakrishnan^3^, Shelley Perkins^3^, Dominic Esposito^3^, Eunkeu Oh^4^, Kimihiro Susumu^4,5^, Mason Wolak^4^, Marc Ferrer^1^_,_ Matthew D. Hall^1^*, Mario J. Borgnia^2^*, and Anton Simeonov^1^*

^1^ National Center for Advancing Translational Sciences, National Institutes of Health, Rockville, Maryland, USA.

^2^ Genome Integrity and Structural Biology Laboratory, National Institute of Environmental Health Sciences, National Institutes of Health, Department of Health and Human Services, Research Triangle Park, NC, USA.

^3^ Protein Expression Laboratory, NCI RAS Initiative, Cancer Research Technology Program, Frederick National Laboratory for Cancer Research, Frederick, MD, USA.

^4^ Optical Sciences Division, Code 5600, Naval Research Laboratory, Washington, D.C., USA.

^5^ Jacobs Corporation, Hanover, Maryland, USA.

*Corresponding author Email: hallma@mail.nih.gov, Mario.Borgnia2@nih.gov, asimeono@mail.nih.gov

^¶^These authors contributed equally: Ying Fu, Juliana da Fonseca Rezende e Mello, Bryan D Fleming and Alex Renn

**Supplemental Material contents:**

Methods: SDS-PAGE and BLI

## SDS-PAGE

## Nanobodies were prepared at 4.5 µg/30 µl and loaded in a 4-20% Tris-glycine gel (Invitrogen). Novex prestained protein ladder was used and loaded at 15 µl per well. Proteins were separated for 25 minutes at 100V, then 60 minutes at 150V. Gel was stained with Imperial protein stain for 45 minutes, then destained in water. Gel was imaged on a BioRad ChemiDoc.

## SARS-CoV-2 mutant S1 and ECD binding (BLI)

Determination of RBD-1-2G-Fc ability to bind mutant S1 and ECD variants was determine by loading anti-human IgG Capture (AHC) biosensor with RBD-1-2G-Fc, then exposing them to various S1 or ECD modalities at 200 nM. RBD-1-2G-Fc was prepared at 10 µg/ml in kinetics buffer (PBS pH 7.4 + 0.02% tween and 0.1% protease-free BSA). Wildtype S1-His (Sino Biological, 40591-V08H), Alpha/B.1.1.7 S1-His (Sino Biological, 40591-V08H12), Beta/B.1.351 S1-His (Sino Biological, 40591-V08H10), Delta/B.1.617.2 S1-His (Sino Biological, 40591-V08H23), Omicron/B.1.1.529 S1-His (Sino Biological, 40591-V08H41), Mu/B.1.621 S1-His (Sino Biological, 40591-V08H38) and Lambda/C.37 S1-His (Sino Biological, 40591-V08H32) were prepared at 200 nM in in kinetic buffer (PBS pH 7.4 + 0.02% tween and 0.1% protease-free BSA). Additionally, Wildtype extracellular domain-His (Sino Biological, 40589-V08B1), Omicron/B.1.1.529 extracellular domain-His (Sino Biological, 40589-V08H26), Omicron/BA.2 extracellular domain-His (Sino Biological, 40589-V08H28) and Lambda/C.37 extracellular domain-His (Sino Biological, 40589-V08H24) were prepared at 200 nM in in kinetic buffer. Biosensors were hydrated for 10 min in kinetics buffer before use. The assay plate was preincubated for 10 min at 30°C before the experiment started. Experimental parameters were baseline 60 sec in kinetics buffer, conditioning 20 sec in 10 mM Glycine (pH 1.5), neutralization 20 sec in kinetics buffer, (condition and neutralization performed 3 times each), baseline 60 sec in kinetics buffer, loading 60 sec, baseline 60 sec, association 300 sec, dissociation 600 sec. For data analysis, the no analyte controls were subtracted from the biosensors readings with analyte. Data were presented as the response value observed at the end of the association phages (300 sec).
