# Supplementary figures and images for: A humanized nanobody phage display library yields potent binders of SARS CoV-2 spike
Source: PLoS One. 2022 Aug 10;17(8):e0272364. doi: 10.1371/journal.pone.0272364 (PMC9365158; doi:10.1371/journal.pone.0272364)

**Figure. S1A**

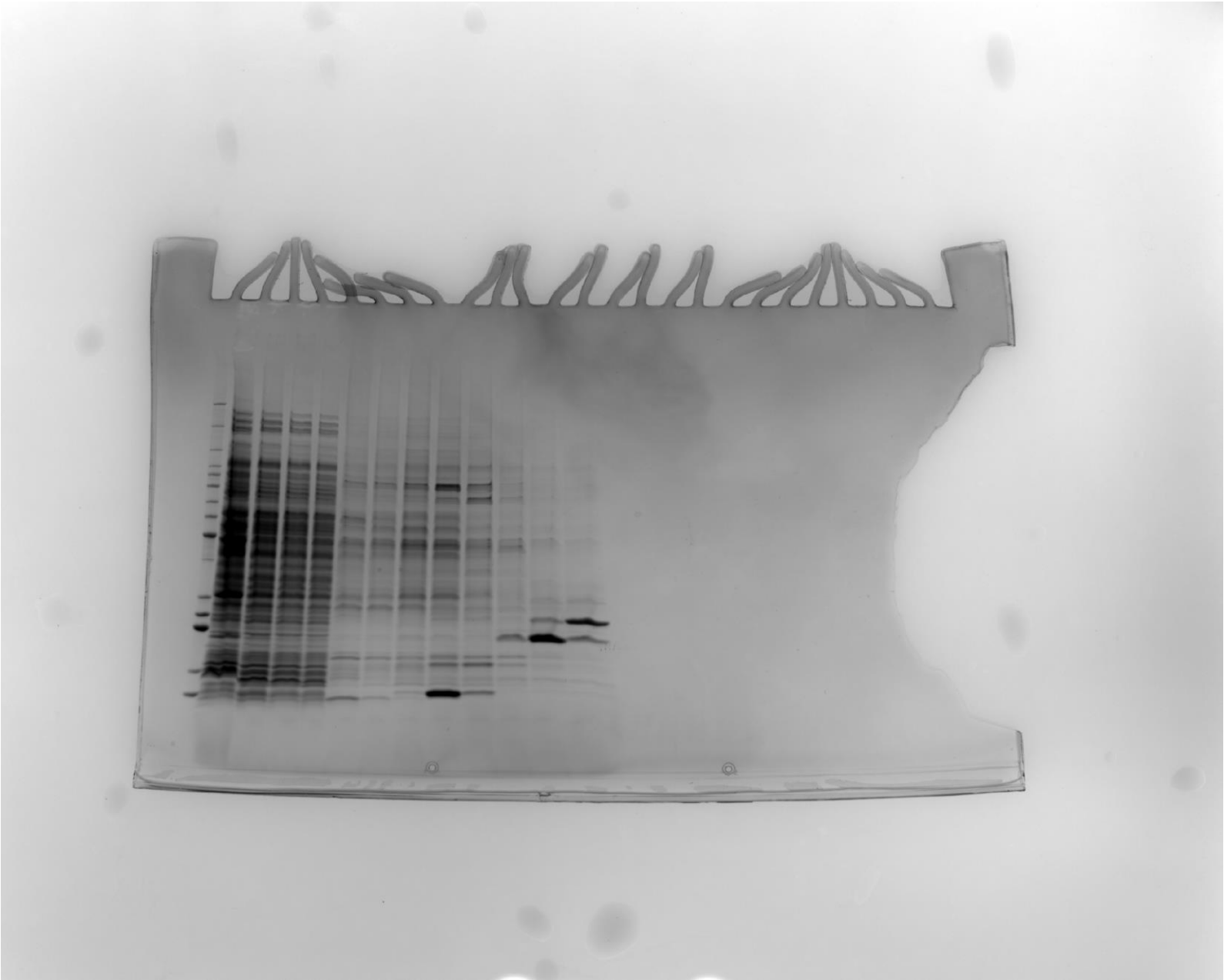

**Figure. S1B**

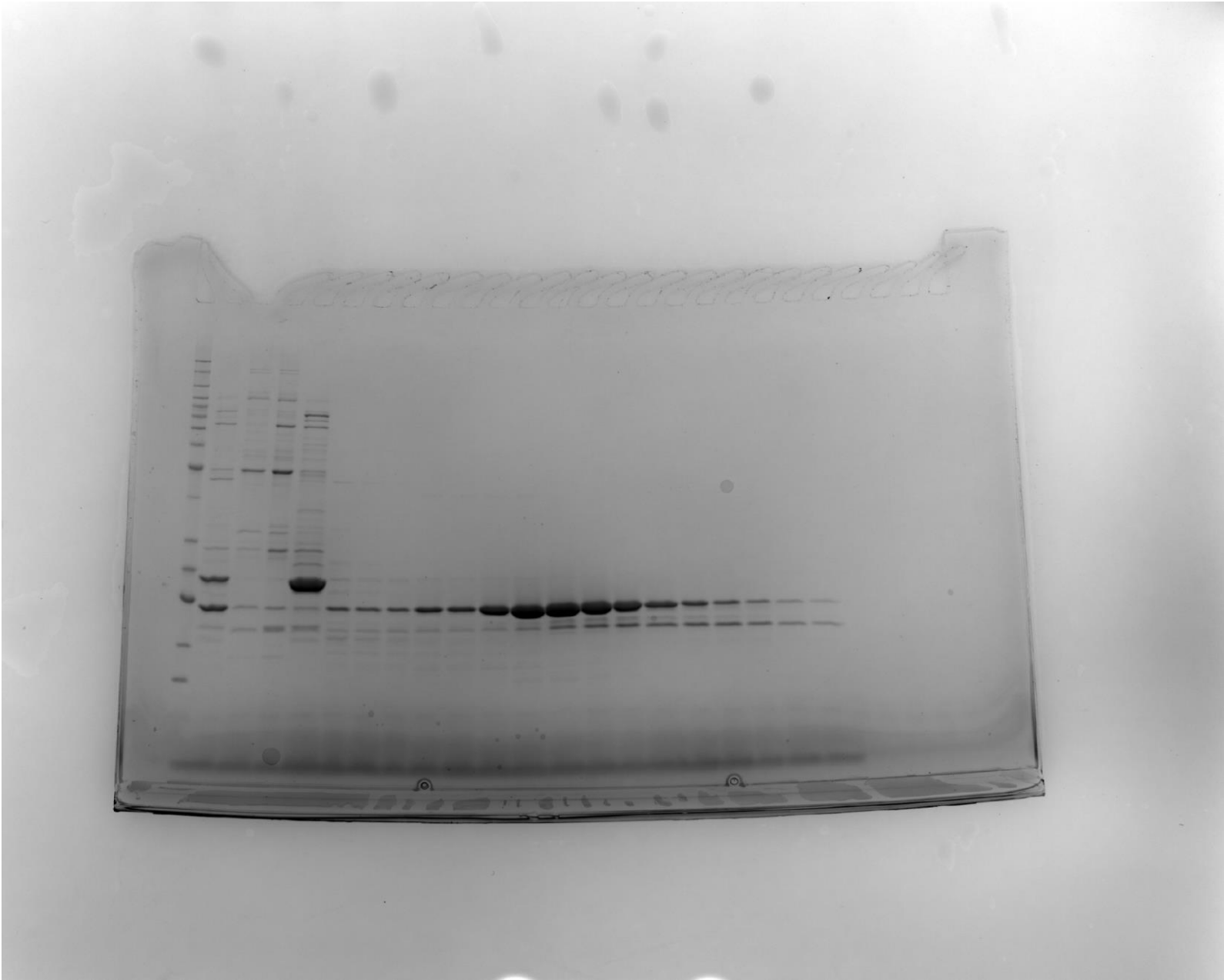

**Figure. S1C**

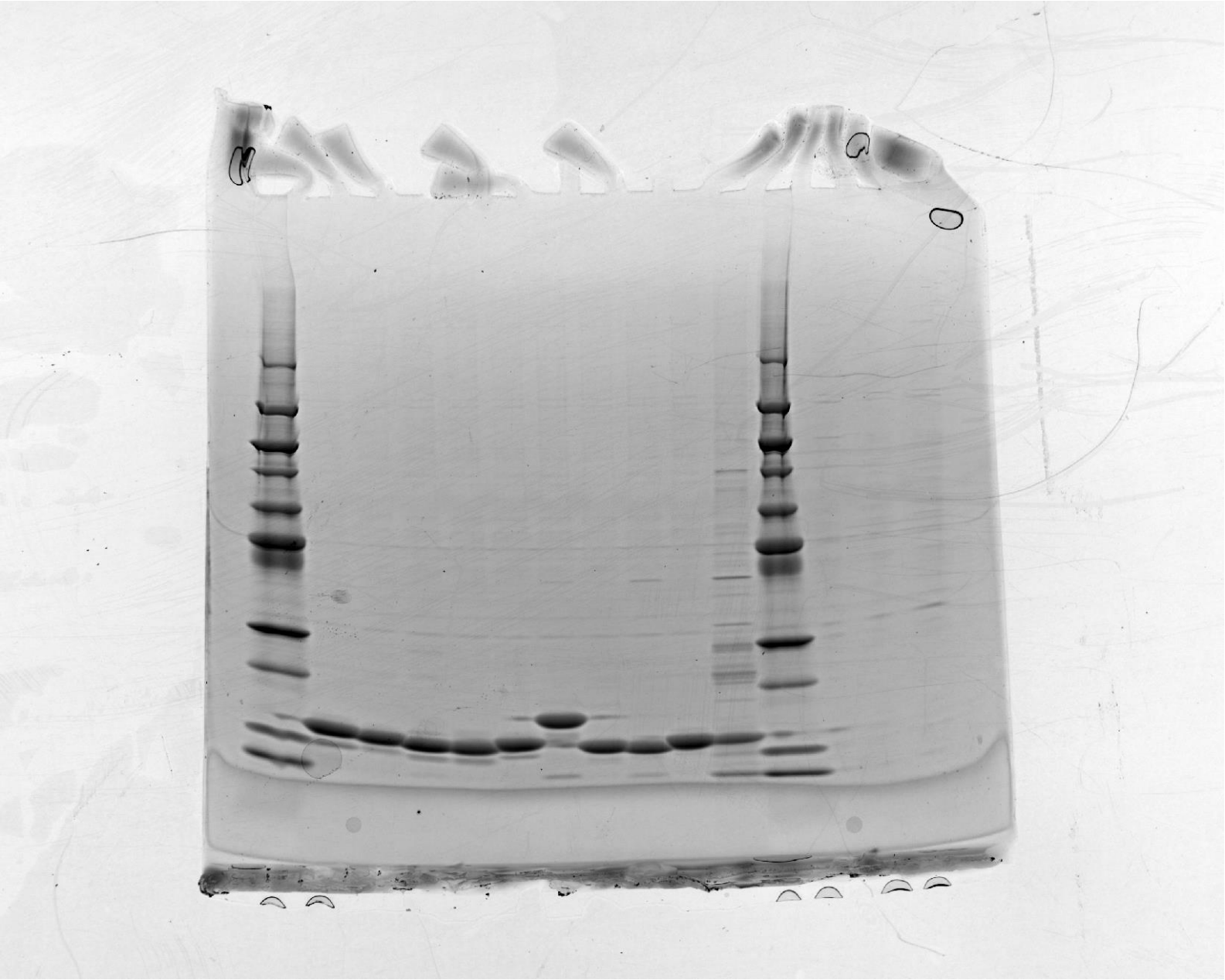

Supplement: S1 File — (PDF) [file pone.0272364.s023.pdf]
